# Cocaine

**Published:** 1890-02

**Authors:** 


### Cocaine.

Dr. Szumann, in the *Therapeutische Monatshefte*, suggests some limits within which cocaine may be used with safety, a point of great practical interest. With doses for adults of one-half to three-fourths to one grain subcutaneously he saw no untoward effects; one grain, however, is adapted only to robust individuals. Some patients will, of course, bear very large doses, among them certain opium takers, who have used as much as seven to thirty-seven grains daily, though one may get toxic symptoms in such patients from an injection of one and a quarter grains.

With nervous patients, and also with those having cardiac disease, as well as those inclined to cerebral congestion, large doses of cocaine may prove dangerous on account of the marked effect on the heart and circulation. Where injections of cocaine are made about the head, not more than half a grain should be used.—*Boston Medical and Surgical Journal*, Nov. 14, 1889.

